# CAREGIVER DEPRESSIVE SYMPTOM SEVERITY AMONG OLDER ADULTS WITH COGNITIVE VULNERABILITY

**DOI:** 10.1093/geroni/igz038.1416

**Published:** 2019-11-08

**Authors:** Richard H Fortinsky, Dorothy Wakefield

**Affiliations:** 1 University of Connecticut School of Medicine, Farmington, Connecticut, United States; 2 UCONN Center on Aging, Farmington, Connecticut, United States

## Abstract

While caregivers of older adults with dementia often report considerable levels of depressive symptoms, much less is known about depressive symptoms among family members of older adults with depression or recent delirium. As part of an ongoing randomized clinical trial testing an in-home multidisciplinary team intervention for older adults with cognitive vulnerability due to dementia, depression, and/or delirium (care recipients, or CR) and their caregivers, in this presentation we report baseline data from the first 211 dyads enrolled in the trial to determine how caregiver depressive symptom severity is related to: CR diagnoses; CR cognitive impairment severity; and CR depressive symptom severity. CR diagnostic groups: Depression Only (n=49); Dementia Only (n=61); Depression and Dementia Only (n=47); Delirium Plus (n=54). Depressive symptom severity was measured using the Center for Epidemiologic Studies Depression Scale; CR cognitive symptom severity was measured using the Telephone Interview for Cognitive Status. Among CR, 57% were female, mean/sd age=77/6.9, 93% White; among caregivers, 64% were female, mean/sd age=66/13.7, 91% White, 55% spouses, 25% daughters, 9% sons. In multivariate linear regression models, which included covariates caregiver gender, relationship to CR, and number of hours/week providing care, we found that caregiver depressive symptom severity was less severe among caregivers of CR with Dementia Only compared to CR with Depression Only (b=-3.32; p=0.06); not associated with CR cognitive symptom severity; and significantly associated with CR depressive symptom severity (b=0.14; p<0.01). We conclude that family members of older adults with depression deserve greater attention to address their own depressive symptoms.

